# The culture of primary duck endothelial cells for the study of avian influenza

**DOI:** 10.1186/s12866-018-1307-4

**Published:** 2018-10-19

**Authors:** Raissa L. Davis, Geunho Choi, Thijs Kuiken, Pascale Quéré, Sascha Trapp, Kirsty R. Short, Mathilde Richard

**Affiliations:** 1 Department of Viroscience, Erasmus Medical Centre, Rotterdam, the Netherlands; 2 INRA ISP, Université de Tours, UMR 1282, Nouzilly, France; 3 School of Chemistry and Molecular Biosciences, University of Queensland, Brisbane, QLD Australia; 4 Australian Infectious Diseases Research Centre, The University of Queensland, Brisbane, QLD 4072 Australia

**Keywords:** Duck, Endothelial cells, Highly pathogenic avian influenza virus

## Abstract

**Background:**

Endothelial cells play a major role in highly pathogenic avian influenza (HPAI) virus pathogenesis in gallinaceous poultry species (e.g. chicken, turkey and quail). Upon infection of gallinaceous poultry with HPAI viruses, endothelial cells throughout the body become rapidly infected, leading to systemic dissemination of the virus, disseminated intravascular coagulation, oedema and haemorrhaging. In contrast, the pathogenesis of HPAI viruses in most wild bird species (e.g. duck, goose and gull species) is not associated with endothelial tropism. Indeed, viral antigen is not found in the endothelial cells of most wild bird species following infection with HPAI viruses. This differential endothelial cell tropism in avian species is poorly understood, mainly due to the absence of appropriate cell culture systems.

**Results:**

Here, we describe the isolation and purification of primary duck endothelial cells from the aorta or bone marrow of Pekin duck embryos. Cells were differentiated in the presence of vascular endothelial growth factor and, if needed, enriched via fluorescent-activated cell sorting based on the uptake of acetylated low-density lipoprotein. The expression of von Willebrand factor, a key marker of endothelial cells, was confirmed by polymerase chain reaction. Monocultures of duck endothelial cells, either derived from the aorta or the bone marrow, were susceptible to infection with an H5N1 HPAI virus but to a much lesser extent than chicken endothelial cells.

**Conclusions:**

The methods described herein to isolate and purify duck endothelial cells from the aorta or bone marrow could also be applied to obtain microvascular endothelial cells from other tissues and organs, such as the lung or the intestine, and represent a valuable tool to study the pathogenesis of avian viruses.

## Background

Influenza A virus represents a significant threat to domestic and wild bird populations. In gallinaceous poultry, most influenza A viruses cause a mild or subclinical infection and are thus referred to as low pathogenic avian influenza (LPAI) viruses. However, some LPAI viruses can evolve in gallinaceous poultry to become highly pathogenic avian influenza (HPAI) viruses. Unlike LPAI viruses, HPAI viruses have a marked tropism for endothelial cells in terrestrial poultry [1]. This endothelial tropism has been associated with disseminated intravascular coagulation, impaired thermoregulation, oedema and haemorrhaging, profuse inflammatory cell recruitment and endothelial cell apoptosis [1]. Accordingly, HPAI in gallinaceous poultry is typically a fatal infection [1]. HPAI viruses can spread from gallinaceous poultry to a wide variety of other avian species. The susceptibility of other bird species to HPAI is largely dependent upon the bird species and virus strain in question [2–4]. However, it is striking to note that viral antigen is not found in the endothelial cells of wild or domestic ducks, following infection with HPAI H5N1 viruses [1, 3]. These observations raise the intriguing possibility that duck endothelial cells may be inherently resistant to infection with HPAI viruses. However, in order to address this question, it is necessary to develop a robust and high throughput methodology to isolate primary duck endothelial cells.

To date, methodologies aimed at isolating primary avian endothelial cells have focussed on those derived from the chicken. Such methodologies include isolating endothelial cells from chicken fat cells [5] and the aortas of both adult birds and chicken embryos [6, 7]. More recently, endothelial cells have been differentiated from endothelial progenitor cells (EPCs) isolated from the blood or bone marrow of chickens [8, 9]. EPCs refer to a heterogeneous cell population that play a key role in the regeneration of endothelial cells that line blood vessels. Bai and colleagues [9] showed that the addition of vascular endothelial growth factor (VEGF) to chicken bone marrow-derived cells induced EPC differentiation into CD34^+^VEGFR-2^+^CD133^−^ endothelial cells. However, to the best of our knowledge, the applicability of any of these methods to the isolation of duck endothelial cells has yet to be investigated.

In order to provide a tool to explore the interactions between HPAI viruses and duck endothelial cells, we sought to develop a technique for isolation of primary endothelial cells from embryonated duck eggs, which are more readily accessible than adult birds. Moreover, we sought to use Pekin (*Anas platyrhynchos domesticus*) duck embryos, as they are more readily accessible than mallard (*Anas platyrhynchos*) duck embryos and the pathogenicity of HPAI viruses in these two species is very similar [3].

## Results

### Duck bone marrow-derived endothelial cells display distinct morphological characteristics

**Fig. 1:**
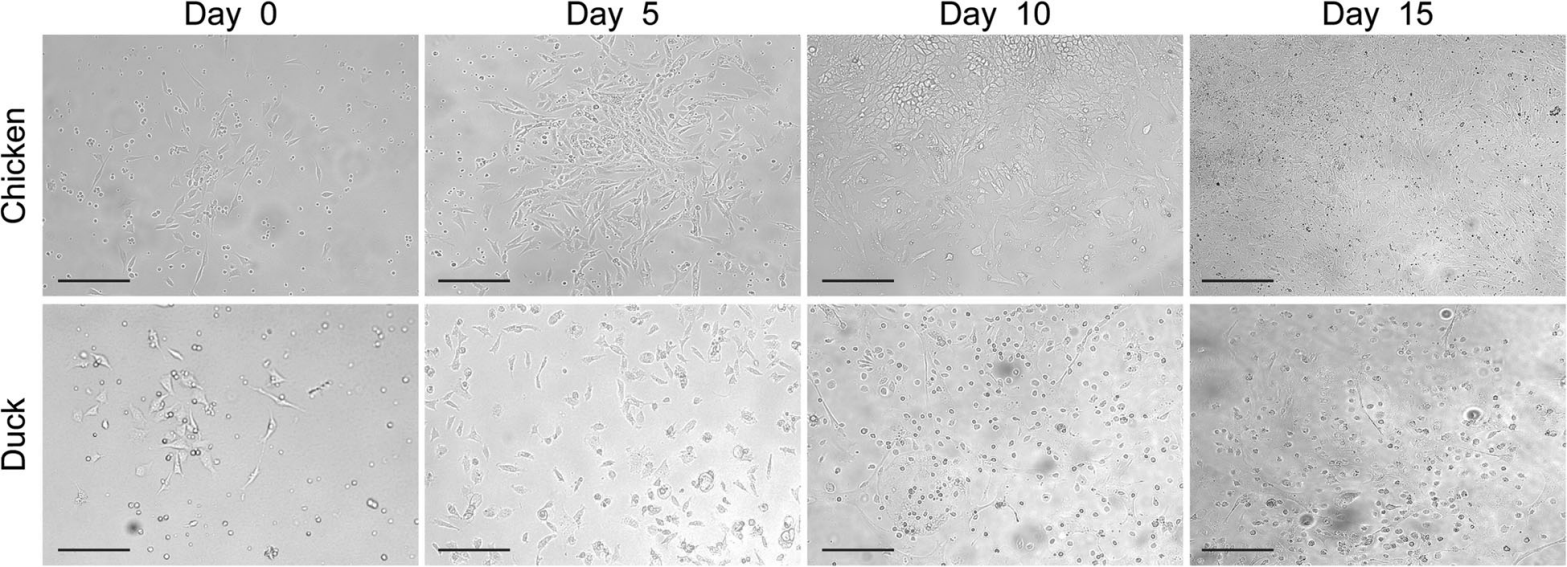
Representative images of chicken and duck bone marrow-derived cells differentiated in the presence of VEGF. Scale bar: 200 nm

Bai and colleagues [9] have previously demonstrated that bone marrow-derived cells from one-day old chickens could be readily differentiated into endothelial cells in the presence of VEGF. We therefore opted to use a similar approach to isolate endothelial cells from duck embryos. Cells were isolated from the bone marrow of 21-day old duck embryos. Cells were then differentiated in endothelial cell growth medium (EGM^TM^-2MV, containing VEGF) for 15 days and the cellular morphology was observed overtime and compared to that of chicken-origin endothelial cells. In contrast to chicken bone marrow-derived cells, most duck bone marrow-derived cells grew as single cells (rather than islands) and were rounder in shape (Fig. 1). It was also possible to observe some elongated cells that formed circular structures (Day 15, Fig. 1).

### Fluorescent-activated cell sorting (FACS) can be used to isolate a pure population of duck endothelial cells

**Fig. 2:**
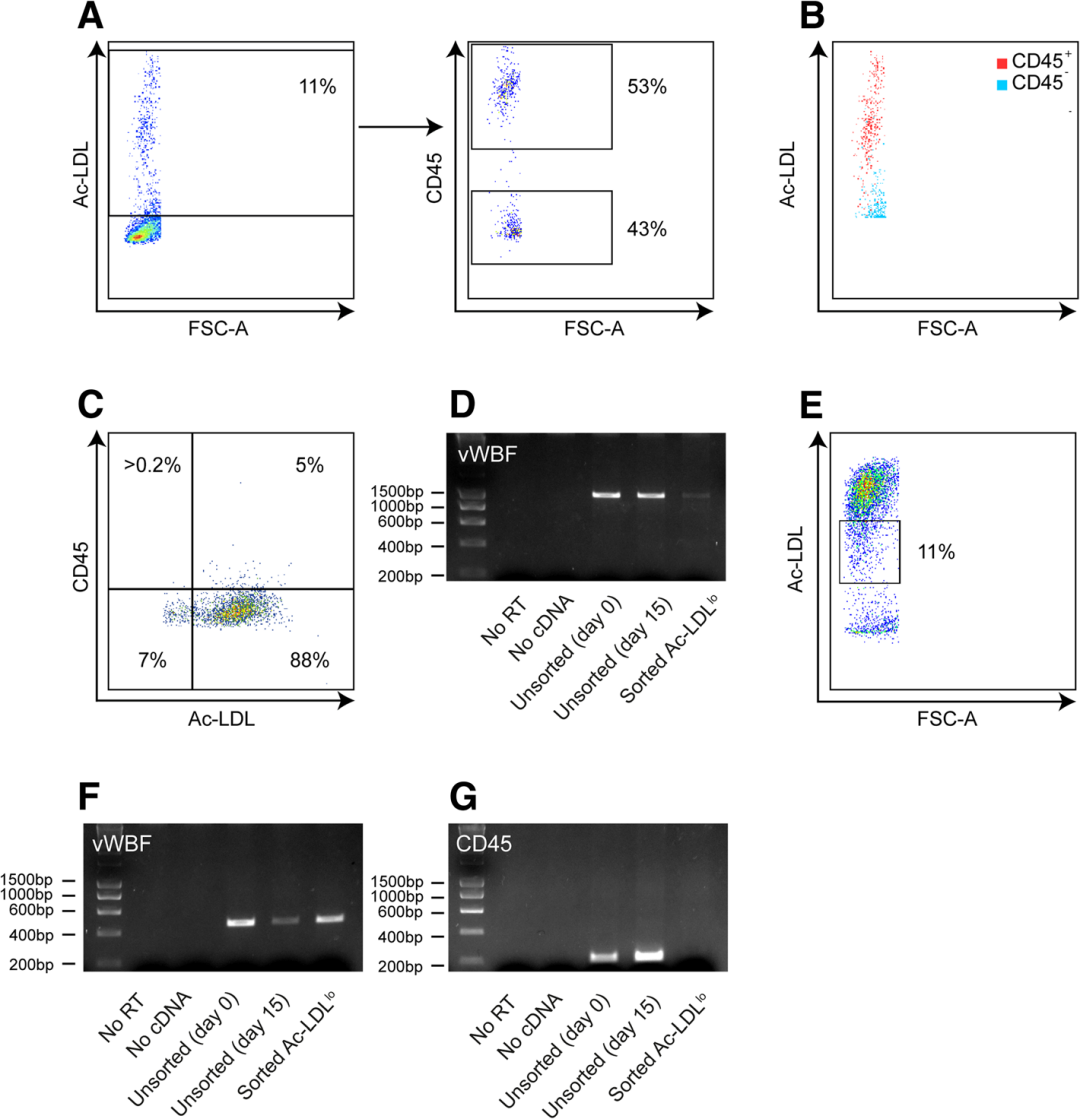
Sorting Ac-LDL^lo^ cells can be used to isolate a pure population of duck endothelial cells. **a**&**b** Representative FACS plots of chicken bone marrow-derived cells following 15 days of culture in human endothelial cell medium. Cells were incubated with Alexa Flour®488 conjugated Ac-LDL for 4 h and then stained for anti-chicken CD45. Single cells (as defined by FCS-A/SSC-A) were assessed for the expression of CD45 and uptake of Ac-LDL. **c** Representative FACS plot of sorted Ac-LDL^lo^ chicken bone marrow-derived cells. Cells were cultured for one passage in human endothelial cell medium, incubated with Alexa Flour®488 conjugated Ac-LDL for 4 h and then stained for anti-chicken CD45. Single cells (as defined by FCS-A/SSC-A) were assessed for the expression of CD45 and uptake of Ac-LDL. **d** RT-PCR for vWF expression on sorted Ac-LDL^lo^ chicken bone marrow-derived cells. ‘No RT’ = samples where RNA was used as the template. **e** Representative FACS plot of sorting strategy used on duck bone marrow-derived single cells following 15 days of culture in human endothelial cell medium. Cells were incubated with Alexa Flour®488 conjugated Ac-LDL for 4 h and then stained for anti-chicken CD45. **f**&**g** RT-PCR for vWF (**f**) and CD45 (**g**) expression on sorted Ac-LDL^lo^ duck bone marrow-derived cells. ‘No RT’ = samples where RNA was used as the template

In order to assess the purity of these cell populations, the uptake of acetylated low density lipoprotein (Ac-LDL) (a feature of both avian and mammalian endothelial cells) [6, 10, 11] was assessed by flow cytometry after the cells were cultured for 15 days in EGM^TM^-2MV medium. Initially, we elected to focus on the purity of the more well-described chicken bone marrow-derived endothelial cells. After 15 days in culture, only 11% of chicken cells were positive for Ac-LDL (Fig. 2a). Whilst Ac-LDL uptake is typically considered to be a specific feature of endothelial cells, it is important to note that Ac-LDL uptake in mammals has also been described for macrophages [12, 13]. Thus, to further determine the purity of the isolated population, chicken Ac-LDL^+^ cells were assessed for the expression of leucocyte common antigen (CD45). CD45 is found on all nucleated cells of hematopoietic origin but not on differentiated endothelial cells [14, 15]. Approximately 50% of Ac-LDL^+^ cells were also positive for CD45 (Fig. 2a). These data suggest that a large proportion of the chicken Ac-LDL^+^ cells were not, in fact, fully differentiated endothelial cells. It is possible to avert this contamination in chickens by performing FACS and only selecting Ac-LDL^+^CD45^−^ cells. However, it is well established that the anti-chicken CD45 antibody necessary for such an approach is not cross reactive with duck leukocytes [16]. Therefore, we sought to develop an alternative approach.

Strikingly, we observed that CD45^+^ chicken bone marrow cells were Ac-LDL^hi^, whilst CD45^−^ bone marrow chicken cells were Ac-LDL^lo^ (Fig. 2b). We thus assessed whether sorting cells on the basis of Ac-LDL expression (Ac-LDL^lo^) was sufficient to eliminate the contaminating CD45^+^ cells. Sorted chicken Ac-LDL^lo^ cells were grown in culture for one passage and then stained for both Ac-LDL and CD45 expression. This sorting strategy resulted in approximately 95% of all cells being Ac-LDL^+^CD45^−^ (Fig. 2c). Moreover, reverse-transcriptase polymerase chain reaction (RT-PCR) showed that sorted endothelial cells remained positive for transcriptional expression of von Willebrand factor (vWF), a key marker of both avian and mammalian endothelial cells [11] (Fig. 2d).

The above data suggest that sorting on Ac-LDL^lo^ cells may be sufficient to generate a pure population of duck endothelial cells. Thus, this sorting strategy was applied to duck bone marrow-derived cells after 15 days in culture with EGM^TM^-2MV medium. Consistent with previous studies [16], the anti-chicken CD45 antibody did not cross-react with duck cells (data not shown). However, it was possible to isolate an Ac-LDL^lo^ population from the duck bone marrow-derived cell cultures (Fig. 2e). To further define the purity of this population, RT-PCR for targeting vWF and CD45 was performed. Whilst the sorted cells remained positive for vWF (Fig. 2f), no CD45 expression could be detected by RT-PCR, suggesting absence of haematopoietic contamination (Fig. 2g). Thus, sorting Ac-LDL^lo^ cells can be used to isolate a pure population of duck endothelial cells.

### Endothelial cells can be isolated from the aorta of duck embryos

**Fig. 3:**
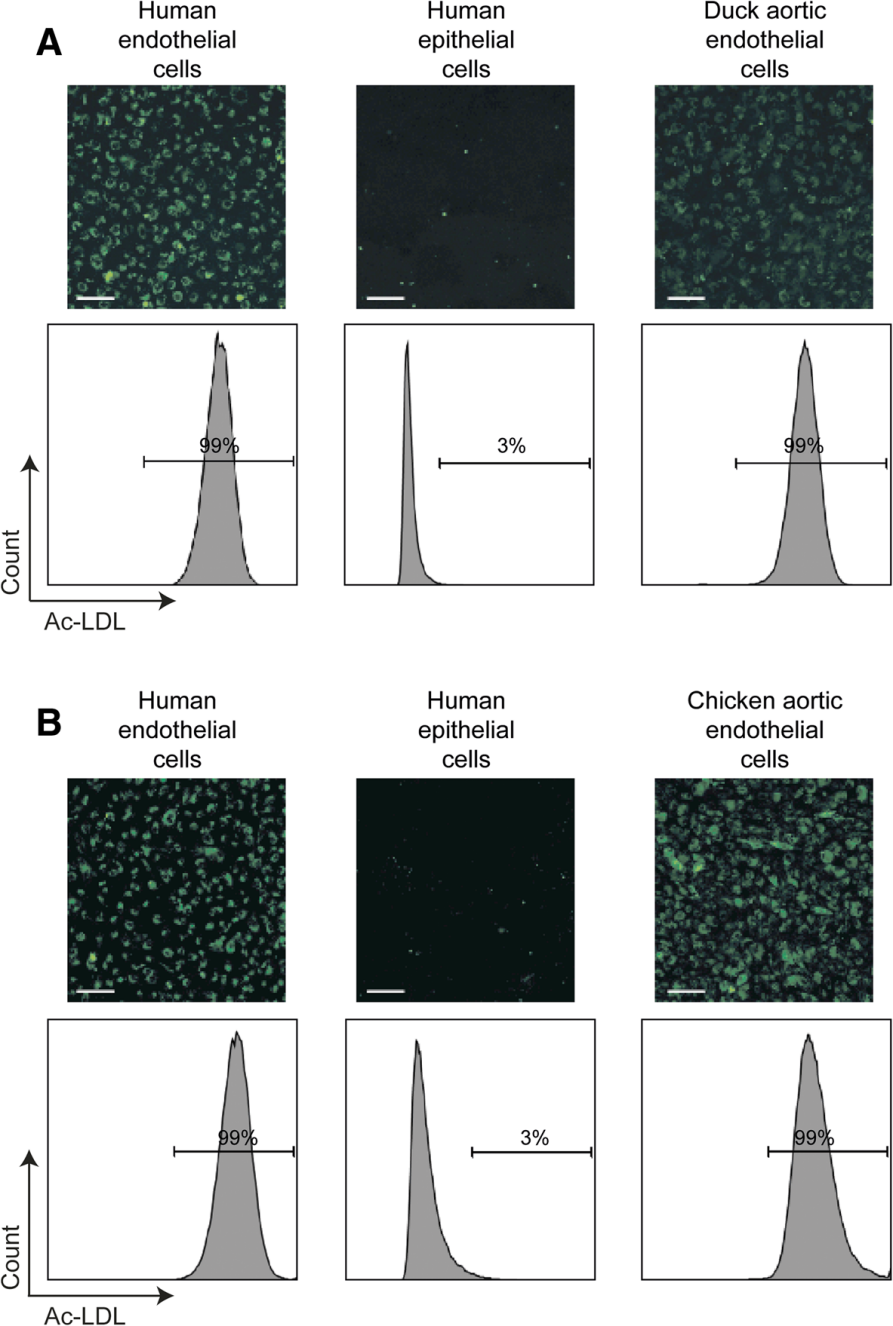
Duck endothelial cells can be isolated from the aorta of embryonated eggs. Representative immunofluorescence images and FACS plots of duck aortic endothelial cells (**a**) and chicken aortic endothelial cells (**b**) following a 4 hour incubation with Alexa Flour®488 conjugated Ac-LDL. Duck and chicken aortic endothelial cells were passaged 15 times and 17 times respectively in EGM^TM^-2MV medium. EA-hy926 and NCl-H441 cells were used as positive and negative control respectively for the uptake of Ac-LDL. Scale bar: 100 nm

Endothelial cells are a heterogeneous population, with function and morphology often being significantly different depending upon the site of isolation [17]. We therefore sought to isolate an alternate population of duck endothelial cells from a different site within the body. We have recently shown that a pure population of endothelial cells can be successfully isolated from the aortas of embryonic chickens [7]. We therefore used the same strategy to isolate duck aortic endothelial cells from embryonated eggs. After 15 passages in EGM^TM^-2MV medium, 99% of cells derived from the duck aortas were Ac-LDL^+^; a percentage comparative to that observed in the human endothelial cell line EA-hy926 (Fig. 3). Flow cytometry on chicken aortic endothelial cells indicated less than 5% of the cell population were CD45+ (data not shown), suggesting that this method was not considerably affected by hematopoietic cell contamination.

### Chicken and duck endothelial cells can be infected in vitro with HPAI virus and are appropriate tools to study the species-dependent pathogenesis of HPAI viruses

**Fig. 4:**
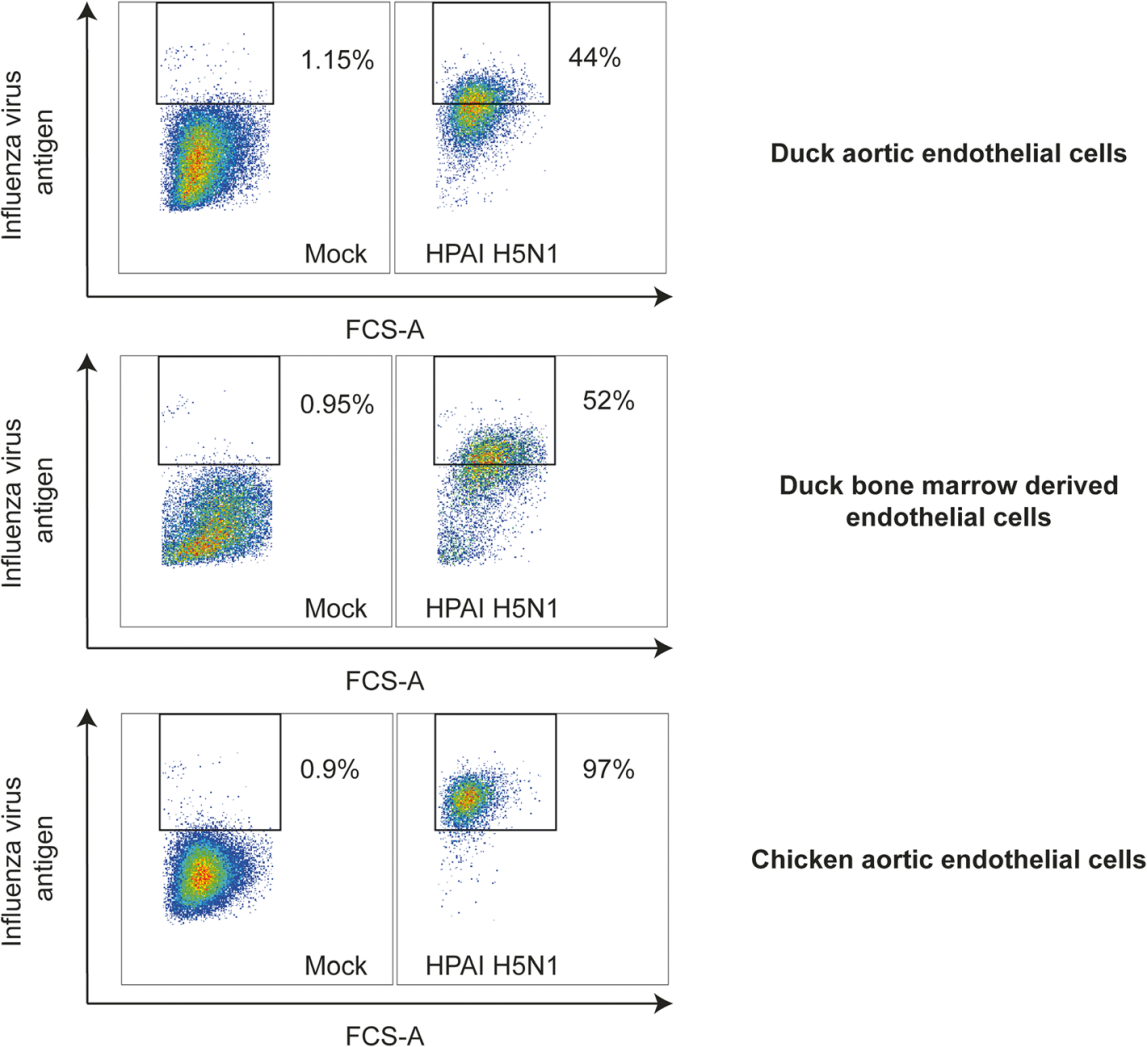
Chicken endothelial cells are more infected than duck endothelial cells by HPAI virus infection. Representative FACS plots showing the number of cells positive for viral antigen 24 h after inoculation with A/turkey/Turkey/1/05 (H5N1). Cells were initially gated on their FSC-A/SSC-A profile. Mock infected cells of each species/cell subtype were then used to define the position of the relevant influenza virus antigen positive gate

Having developed two techniques to isolate primary endothelial cells from ducks, we next wished to determine if these cell cultures would be appropriate tools to use to understand species-dependent differences in HPAI virus infection. Thus, primary duck aortic endothelial cells, primary duck bone marrow-derived endothelial cells and chicken aortic endothelial cells were infected with a HPAI H5N1 virus for 24 h and the percentage of infected cells were measured by flow cytometry. Figure 4 shows that, whilst all cell types were successfully infected with HPAI H5N1, the percentage of chicken aortic endothelial cells that were infected with HPAI H5N1 after 24 h was markedly higher than that of both duck aortic endothelial cells and duck bone marrow-derived cells endothelial cells. These data suggest that the isolated endothelial cells are useful tools to study species dependent differences in HPAI virus infections.

## Discussion

In gallinaceous poultry, endothelial cells are an important cellular target of HPAI viruses [1]. In contrast, viral antigen is rarely detected in the endothelial cells of infected mallard and Pekin ducks [1]. Our understanding of these species-dependent differences in viral tropism has been significantly impaired by the lack of techniques available to isolate primary duck endothelial cells. Here, we developed and validated new techniques for isolating primary duck endothelial cells in order to gain a new insight into the pathogenesis of avian influenza viruses.

Both techniques used in the present study were based upon those previously described for isolating primary chicken endothelial cells [7, 9]. Interestingly, in the present study we had to adapt the protocol of Bai and colleagues [9] in order to prevent CD45+ cells contaminating chicken endothelial cell cultures. The necessity for this step may reflect the fact that whilst Bai and colleagues [9] isolated bone marrow cells from fully developed birds, we used those derived from avian embryos (which may contain a higher proportion of Ac-LDL+ CD45+ cells). Although we cannot eliminate the possibility that embryo-derived endothelial cells have different phenotypic or functional characteristics compared to adult endothelial cells, the use of embryos represents a cheaper and more accessible option for the preparation of primary cell cultures.

Previous in vivo studies have suggested that duck endothelial cells are refractory to HPAI virus infection [1]. However, in the present in vitro study we found that duck endothelial cells (both bone marrow-derived and aortic) were, to a certain extent, susceptible. This suggests that some additional factor that was missing from the established in vitro culture system (e.g. another cell type or soluble factor) which may serve to further limit infection in vivo. We recently showed that human endothelial cells did not become infected in an in vitro co-culture system of human endothelial and epithelial cells [18] despite the fact that monocultures of human endothelial cells were susceptible to infection. Thus, our data may reflect the fact that direct in vitro infection of duck endothelial cells does not represent the in vivo situation, whereby viruses have to pass through the epithelium to reach the endothelium. Nevertheless, both duck endothelial cell cultures were less infected than chicken aortic cells when cell cultures when examined 24 h after infection. These differences in infection rate could be associated with species-dependent differences in protease expression, such that HPAI viruses are unable to undergo multi-cycle growth in duck endothelial cells, or species-specific innate immune restrictions. Additional experiments using LPAI and HPAI viruses are needed to fully assess the susceptibilities of chicken and duck endothelial cells to avian influenza viruses.

## Conclusions

The primary avian endothelial cell cultures developed herein are a valuable tool to further dissect species dependent differences in viral tropism. Indeed, these culture systems also afford the possibility to better study the pathogenesis of other infectious diseases of ducks caused by endotheliotropic viruses (such as duck enteritis virus) [19]. Moreover, the cell-sorting technique described in this article for the isolation of bone marrow-derived endothelial cells could also be applied to endothelial cells from other tissues and organ sites, such as respiratory tract and intestinal mucosal surfaces and the blood-brain barrier. The continued development of these and other robust techniques for primary avian cell culture remains essential for our understanding of avian infectious diseases.

## Methods

### Cells

EA-hy926 (human umbilical vein endothelial cells) and NCl-H441 cells (human papillary adenocarcinoma lung epithelial cells) were purchased from the ATCC (CRL-2922 and HTB-174 respectively). These human cell lines were maintained in Roswell Park Memorial Institute (RPMI, Lonza) with 10% foetal calf serum (FCS) (Greiner) and 100 U/ml penicillin (Lonza), 100 U/ml streptomycin (Lonza) in a humidified 37 °C incubator with 5% carbon dioxide (CO_2_). Madin-Darby canine kidney cells (MDCK, ATCC CCL-34) were cultured in Eagle’s minimal essential medium (EMEM, Lonza) supplemented with 10% FCS (Greiner), 100 U/ml penicillin, 100 U/ml streptomycin, 2 mM L-glutamine (Lonza), 1.5 mg/ml sodium bicarbonate (NaHCO_3_, Lonza), 10 mM Hepes (Lonza) and 1X non-essential amino acids (NEAA, Lonza). 293T cells (ATCC CRL-3216) were cultured in Dulbecco modified Eagle’s medium (DMEM, Lonza) supplemented with 10% FCS, 100 U/ml penicillin, 100 U/ml streptomycin, 2 mM L-glutamine, 1 mM sodium pyruvate (ThermoFisher scientific) and 1X NEAA.

### Isolation of chicken and duck bone marrow-derived cells

Eighteen day-old embryonated chicken (*Gallus Gallus domesticus*) eggs and twenty-one day-old Pekin duck (*Anas platyrhynchos domesticus*) eggs were cold-anesthesized at 4 °C for 15 min. Embryos were euthanised by decapitation, dissected under sterile conditions and femur and tibiotarsus bones were collected in DMEM medium. Ends of the bones were cut using scissors to expose the bone marrow cavity. The bone marrow cavity was flushed with pre-chilled DMEM medium using a 10 ml syringe with a 0.5 × 16 mm needle and bone marrow cells were collected in a 50 ml tube placed on ice. Bone marrow cells were filtered through a 40 μm cell strainer and were collected in 50 ml tubes. Bone marrow cells were centrifuged at 300 *g* for 5 min at 4 °C and resuspended in DMEM medium. Fifteen ml of bone marrow cell suspension was carefully layered over 15 ml of Lymphoprep™ (Stemcell Technologies) and subsequently centrifuged at 300 *g* for 20 min at 4 °C with no break. The cell layer at the interface between the Lymphoprep™ and medium was collected using a Pasteur pipette and diluted in 5 ml of DMEM medium. The cell suspension was centrifuged at 300 *g* for 5 min at 4 °C. After centrifugation, cells were resuspended in 1 ml of EGM^TM^-2MV (Lonza) and viable cells were enumerated using a Trypan Blue staining. Finally 1.5 × 10^6^ viable cells were plated on 0.2% gelatin (Sigma-Aldrich) coated culture dish containing 10 ml EGM^TM^-2MV medium and incubated at 37 °C, 5% CO_2_. EGM^TM^-2MV medium was refreshed every 3 to 4 days. On some occasions, cells were cryopreserved in 90% FCS-10% dimethyl sulfoxide (DMSO) and thawed for FACS.

### FACS of bone marrow-derived endothelial cells

After 15 days in culture, chicken and duck bone marrow-derived cells were used for sorting. Bone marrow-derived cells were incubated for 4 h in EGM^TM^-2MV medium containing 3.3 μg/ml of Alexa Fluor®488 conjugated Ac-LDL (ThermoFisher Scientific). Bone marrow-derived cells were then washed with phosphate-buffered saline (PBS) and treated with 0.05% trypsin-Ethylenediaminetetraacetic acid (EDTA) (ThermoFisher Scientific). Dissociated bone marrow-derived cells were moved to a 50 ml tube and diluted with 20 ml of RPMI medium with 10% FCS. The cell suspension was centrifuged at 300 *g* for 5 min and resuspended with 1 ml of PBS with 2% FCS. Where relevant, 10^6^ bone marrow-derived cells were stained with 10 μg/ml of monoclonal mouse Immunoglobulin G (IgG) anti-chicken CD45 (Bio-Rad) diluted in PBS with 2% FCS for 20 min at 4 °C. Cells were washed twice with PBS with 2% FCS. Antigen expression was revealed by staining with 20 μg/ml of Allophycocyanin (APC) conjugated goat anti-mouse IgG antibody (BD Biosciences) diluted in PBS with 2% FCS for 20 min at 4 °C. Cells were washed twice and with PBS with 2% FCS. FACS was performed using a BD FACSCanto II (BD Biosciences). Flow cytometry analysis was performed using FlowJo version 8.8.7 (TreeStar, Inc.). Sorted cells were plated in a well of a 48-well plate (20,000 cells/well) coated with 0.2% gelatin and were incubated in EGM^TM^-2MV medium at 37 °C, 5% CO_2_. EGM^TM^-2MV medium was changed every 3 to 4 days. Cells were passaged when confluence was reached.

### Isolation of chicken and duck aortic endothelial cells

Isolation of chicken and duck aortic endothelial cells was performed as previously described [7]. Eighteen day-old embryonated chicken eggs and 21 day-old embryonated duck eggs were cold-anesthesized at 4 °C for 15 minutes. Embryos were euthanised by decapitation and dissected under sterile conditions. Hearts were harvested in DMEM medium. The ascending aortic arches were carefully separated from the hearts and minced into smaller pieces using scalpels onto a glass plate. These pieces were transferred to a culture dish coated with 0.2% gelatin containing 10 ml of EGM^TM^-2MV medium. Aortic cells were incubated at 40 °C, 5% CO_2_ for 2 days. Two days after isolation, the pieces of aortic arches were carefully washed away with PBS and 10 ml of fresh EGM^TM^-2MV medium were added to the culture dish. Aortic cells were passaged every 3 to 4 days. Aortic cells were passaged for a minimum of 15 times and cryopreserved on some occasions in 90% FCS-10% DMSO.

### Ac-LDL uptake of aortic endothelial cells

Chicken and duck aortic endothelial cells were incubated for 4 h in EGM-2MV medium containing 3.3 μg/ml of Alexa Fluor®488 conjugated Ac-LDL (ThermoFisher Scientific). Cells were then washed with medium without Alexa Fluor®488 conjugated Ac-LDL and were visualised using a Laser Scanning Microscope 700 (LSM 700) (Zeiss, Jena, Germany).

### Expression of vWF and CD45 encoding genes detected by RT-PCR

Ribonucleic acid (RNA) was isolated with RNeasy Mini Kit (Qiagen) according to the manufacturer’s instructions. RNA was reverse transcribed using random primers (Promega) and SuperScript III reverse transcriptase (ThermoFisher Scientific). In order to ensure that there were no contamination with genomic deoxyribonucleic acid (DNA) in the extracted RNA, 100 ng of extracted RNA was also used as a template for polymerase chain reaction (PCR). The PCR mix contained 1 μl of *PfuUltra* II DNA polymerase (Agilent Technologies), 5 μl of 10X PCR buffer, deoxynucleotide triphosphate (dNTP) Mix (18 mmol each, Roche Diagnostics), 2 μg template complementary DNA (cDNA), 5 μl of forward primer and 5 μl of reverse primer (2 pmol/μl) in a total volume of 50 μl. PCR for vWF messenger RNA (mRNA) amplification was performed with the following conditions: (i) denaturation for 3 minutes (min) at 95 °C, (ii) the first 25 cycles of amplification with denaturation for 1 min at 95 °C, annealing for 30 seconds (s) at 60 °C and extension for 1 min at 72 °C, (iii) the next 15 cycles of amplification with denaturation for 1 min at 95 °C, annealing for 30 s at 40 °C and extension for 1 min at 72 °C, (iv) final extension for 10 min at 72 °C. PCR for CD45 mRNA amplification was performed with the following conditions: (i) denaturation for 3 min at 95 °C, (ii) 30 cycles of amplification with denaturation for 1 min at 95 °C, annealing for 30 s at 50 °C and extension for 1 min at 72 °C, (iii) final extension for 10 min at 72 °C. The primer sequences are listed in Table 1. PCR products were visualized on a 1% agarose gel and pictures were taken using the ChemiDoc XRS+ (Bio-rad). PCR bands were extracted from the agarose gel and purified using the QIAquick Gel extraction kit (Qiagen) according to the manufacturer’s instructions and were sequenced using the BigDye Terminator v3.1 Cycle sequencing kit (Applied Biosystems) and the 3130XL genetic analyser (Applied Biosystems). The sequences obtained from the PCR products were compared with those obtained from Genbank (chicken vWF, accession number BK007988.1, duck vWF, accession number XM_005012640.3 and duck CD45, accession number XM_021277405.1).

**Table 1:** Primer sequences

Host	Name	Sequence
Chicken	vWF	Forward: GCCAATGACTTCATG Reverse: GCCACAGTCATTGGTG
Duck	vWF	Forward: ACCACATGTTAGTGAGGAAC Reverse: CTTGGTAGGGTATGCTTCTC
	CD45	Forward: ATTGCCAGTATCTACCCTGC
		Reverse: TGTTGAGCTTTCTGTTCCCT

### Infection of chicken and duck endothelial cells with HPAI viruses

Wild-type virus isolate A/turkey/Turkey/1/05 (HPAI, H5N1) was obtained from the World Health Organization (WHO) Collaborating Center in London. It was grown in MDCK cells and embryonated chicken eggs and titred by end-point titration in MDCK cells. Endothelial cells were infected at a multiplicity of infection (MOI) of 1 at 37 °C, 5% CO_2_ (bone marrow-derived endothelial cells) and at 40 °C, 5% CO_2_ (aortic endothelial cells). At 24 h post-infection, cells were trypsinised and pelleted in a 96-well plate for staining for the influenza A virus nucleoprotein (NP) as a marker for infection. Cells were fixed and permeabilized with BD Cytofix/Cytoperm™ (BD Biosciences). Cells were then washed twice with the BD Perm/Wash™ buffer (BD Biosciences) and subsequently stained with anti-influenza NP antibody (HB-65, ATCC) diluted in BD Perm/Wash™ buffer. Cells were then washed twice with the BD Perm/Wash™ buffer and subsequently stained with 10μg/ml of Alexa Fluor®488 conjugated goat anti-mouse IgG2a antibody (ThermoFisher Scientific) diluted in BD Perm/Wash™ buffer. Cells were then analysed by flow cytometry using a BD FACSCanto II cytometer (BD Bioscience). Flow cytometry analysis was performed using FlowJo version 8.8.7 (TreeStar, Inc.). Experiments were carried out under biosafety level 3 (BSL3) conditions.

## Abbreviations

Ac-LDL: Acetylated low density lipoprotein
APC: Allophycocyanin
BSL3: Biosafety level 3
CD45: Leucocyte common antigen
cDNA: Complementary deoxyribonucleic acid
CO_2_: Carbon dioxide
DMEM: Dulbecco modified Eagle’s medium
DMSO: Dimethyl sulfoxide
DNA: Deoxyribonucleic acid
dNTP: Deoxynucleotide triphosphate
EA-hy926: Human umbilical vein endothelial cells
EDTA: Ethylenediaminetetraacetic acid
EGM^TM^-2MV: Endothelial cell growth medium
EMEM: Eagle’s minimal essential medium
EPC: Endothelial progenitor cells
FACS: Fluorescent-activated cell sorting
FCS: Foetal calf serum
HPAI: Highly pathogenic avian influenza virus
IgG: Immunoglobulin G
LPAI: Low pathogenic avian influenza virus
LSM: Laser scanning microscope
MDCK: Madin-Darby canine kidney
min: minute
MOI: Multiplicity of infection
mRNA: Messenger ribonucleic acid
NaHCO_3_: Sodium bicarbonate
NCl-H441: Human papillary adenocarcinoma lung epithelial cells
NEAA: Non-essential amino acids
NP: Nucleoprotein
PBS: Phosphate-buffered saline
PCR: Polymerase chain reaction
RNA: Ribonucleic acid
RPMI: Roswell Park Memorial Institute
RT-PCR: Reverse transcriptase polymerase chain reaction
s: second
VEGF: Vascular endothelial growth factor
vWF: Von Willebrand factor
WHO: World Health Organization
